# Comparative study and relationship analysis between purine content, uric acid, superoxide dismutase, and growth traits in purebred and crossbred Thai native chickens

**DOI:** 10.3389/fvets.2023.1263829

**Published:** 2023-09-25

**Authors:** Veeraya Tantiyasawasdikul, Kitsadee Chomchuen, Wipas Loengbudnark, Vibuntita Chankitisakul, Wuttigrai Boonkum

**Affiliations:** ^1^Department of Animal Science, Faculty of Agriculture, Khon Kaen University, Khon Kaen, Thailand; ^2^Network Center of Animal Breeding and Omics Research, Khon Kaen University, Khon Kaen, Thailand

**Keywords:** body weight, SOD, purine, correlation, indigenous chickens

## Abstract

The objective was to compare and analyze the relationship between growth, purine content, uric acid, and superoxide dismutase (SOD) in purebred and crossbred Thai native chickens. A total of 300 Thai native chickens were divided into 3 groups. Group 1 was purebred Thai native chickens (100%TN), Group 2 was 50% Thai native chickens (50%TN), and Group 3 was 25% Thai native chickens (25%TN). Data included the body weight (BW), average daily gain (ADG), and breast circumference (BrC). At 6, 8, and 10 weeks of age, 10 chickens from each group were randomly euthanized to collect breast meat, liver, and blood samples to analyze the purine content consisting of total purine, adenine, guanine, xanthine, and hypoxanthine, and uric acid, in breast meat and liver and SOD in blood. A general linear model, Pearson correlation and principal component analysis were used to analyze the significant differences and relationship between variables. The results showed the 25%TN group had the highest growth traits at every age, while the 100%TN group had the lowest (*p* < 0.05). Consistent with the analysis results of purine values, purine content and uric acid in breast meat and liver and SOD in blood decreased with age (*p* < 0.05). The correlations between purine content (total purine, adenine, guanine, xanthine, and hypoxanthine) and growth traits (BW, ADG, and BrC) ranged from moderate negative to moderate positive (−0.542 to 0.253)(*p* < 0.05). The correlations between uric acid and growth traits (0.348–0.760) and SOD and growth traits (0.132–0.516) were low to moderate positive with significant differences (*p* < 0.05). The principal component plot, which highlighted three principal components (PC 1, PC 2, and PC 3), explained 86.44 and 86.53% of the total information in breast meat and liver for selecting animals for optimal balance of the variation in the growth traits, purine content, uric acid, and SOD. Although purebred Thai native chickens showed the lowest growth traits, purine content, uric acid, and SOD were also lowest compared to crossbred Thai native chickens. Therefore, the development of genetics in Thai native chickens to produce healthy food could be possible.

## 1. Introduction

Growth characteristics are one of the most economically important traits and have been continuously improved genetically in both commercial strains and native chickens (1–3) to produce more food for the world’s population, which is growing steadily (4, 5). While fast-growing chickens allow farmers to speed up production cycles, the other effect is an increase in undesirable bioactive compounds in the meat, especially increased accumulation of purine content and uric acid levels in meat and organs (6, 7).

Purines are nitrogen-containing compounds in many foods, including meat, seafood, and poultry (8–10). When the body metabolizes purines, they can be broken down into uric acid, accumulating in the blood and tissues and causing health problems, such as gout, hyperuricemia, renal dysfunction, and other cardiovascular diseases (11). Several studies have shown that fast-growing chickens could have higher levels of purines and uric acid in their meat and organs than slow-growing chickens (12, 13), and the levels of purines and uric acid are different from organ to organ, particularly in the liver, which is the most important organ to generate purines and uric acid (14, 15). This is because purines are essential components of deoxyribonucleic acid (DNA) and ribonucleic acid (RNA), which are necessary for cell division and protein synthesis (16, 17). As chickens grow rapidly, their muscle development and other metabolic processes require significant protein synthesis. Purines are essential for forming nucleotides, the building blocks of DNA and RNA, and play a role in energy metabolism (7, 18). However, chickens cannot synthesize purines *de novo* (19, 20); consequently, they must obtain purines through their diet or by recycling purines from other metabolic processes. Therefore, to improve their fast growth rate, chickens require more purines to support their metabolic needs (7). As a result, chickens that grow more quickly may have higher levels of purines in their tissues, which can be transferred to consumers who consume their meat or chicken products (13, 21, 22).

At the same time, fast-growing chickens are more prone to stress than slow-growing chickens due to their rapid growth rate and increased metabolic demands; the consequences are that fast-growing chickens have health and welfare problems (23, 24). In addition, fast-growing chickens are more susceptible to stressors such as overcrowding, high temperatures, and poor air quality (25, 26), which leads to increased mortality rates, reduced immune and antioxidant enzymes [catalase (CAT), superoxide dismutase (SOD), and glutathione peroxidase (GPX)], physiological parameters (27–30), and behavioral problems such as feather pecking and cannibalism (31). In contrast, slow-growing chickens may have a more robust immune system, better adaptability to environmental stressors, and lower susceptibility to behavioral problems (32, 33).

As mentioned above, this aligns with the popularity of consumers, who are more interested in good quality and healthy food (34, 35). Slow-growing chicken meat produced from native chickens is becoming increasingly interesting because it has several distinctive properties compared to commercial broiler meat products, including a unique flavor, firm and tender meat (36, 37), lower fat (38) and high levels of bioactive compounds, such as angiotensin-converting enzyme inhibitors (ACE), anserine, and carnosine, which have health benefits for consumers (13, 39–42).

However, the limitation of the slow growth rate in native chickens compared to commercial strains makes it challenging to raise native chickens for business. For this reason, farmers have used a crossbreeding mating system between commercial strains and native chickens to solve this problem (43–45). In this regard, it is necessary to have academic supporting information, especially the association between body weight traits, purines, uric acid, and SOD in native chicken meat and organs. Therefore, to ensure the development of crossbred native chicken genetics with both commercial potential and good properties of being a healthy food, the aim of this study was to compare and analyze the relationship between growth, purine content, uric acid, and SOD in purebred and crossbred Thai native chickens. Our results will benefit genetic selection in the slow-growing chicken breeding program.

## 2. Materials and methods

### 2.1. Ethics statement and animal management

All animal procedures were approved by the Institutional Animal Care and Use Committee, based on the Ethics of Animal Experimentation of the National Research Council of Thailand (record no. IACUC-KKU-14/65). This study was conducted at the experimental farm of the Network Center for Animal Breeding and Omics Research (NCAB), Faculty of Agriculture, Khon Kaen University, Thailand. A total of 300 Thai native chickens (Shee breed), which have been genetically selected from previous breed improvement program, were divided into 3 groups of 100 birds each. Group 1 was purebred Thai native (TN) chickens (100%TN), Group 2 was 50% female Thai native with 50% male broiler chickens (Ross 308 breed; 50%TN), and Group 3 was 25% female Thai native with 75% male broiler chickens (25%TN). Housing and management were processed under Thai native chicken rearing standards, and all birds were reared in an open-environmental system. The birds were fed *ad libitum* with commercial diets divided into 2 phases: starting phase (21% crude protein, 5% crude fiber, 3,100 kcal of ME/kg) for the first 4 weeks after hatching (0–4 weeks of age) and growing phase (19% crude protein, 5% crude fiber, 3,200 kcal of ME/kg) from 4 weeks old to the end of the experiment (10 weeks of age). When all chicks were born, they were numbered with legs tagged for identification until 4 weeks of age, then they were numbered with wings tagged. All chicken groups were raised using warming with a 100-watt lamp for 2 weeks. The lightening program consisted of two stages: the first stage was from hatching to 4 weeks with 24 h light/0 h dark; the second stage was from 5 to 10 weeks with natural light.

### 2.2. Data collection

Growth traits were recorded for each chicken from all groups. Body weights were individually collected using a weighing scale every 2 weeks from hatch to 10 weeks old. Breast circumferences were individually collected using measuring tape by inserting the tape beneath both wings and measuring the circumference at the largest part of the breast. The growth traits consisted of body weight at hatch (BW0), body weight at 2, 4, 6, 8, and 10 weeks of age (BW2, BW4, BW6, BW8, and BW10), average daily gain (ADG) during 0–2, 2–4, 4–6, 6–8, and 8–10 weeks of age (ADG0–2, ADG2–4, ADG4–6, ADG6–8 and ADG8–10), and breast circumference at 6, 8, and 10 weeks of age (BrC6, BrC8, and BrC10). At 6, 8, and 10 weeks of age, in each chicken group (100%TN, 50%TN, and 25%TN), 10 chickens (5 males and 5 females) were randomly euthanized to collect breast meat, liver, and blood samples. Breast meat samples were randomly collected from 6 locations from both sides of the breast, right side (upper, middle, lower) and left side (upper, middle, lower) using lancets and keeping the samples in plastic bags. Livers were collected for the whole piece and kept in plastic bags. The samples were prepared for the next step. The purine contents adenine, guanine, xanthine, and hypoxanthine, and uric acid (calculated from purine content), in breast meat and liver and superoxide dismutase (SOD) in blood were analyzed. The breast meat and liver were preserved using snap-freezing in liquid nitrogen and stored at −20°C for estimation of purine content and uric acid. Blood samples (approximately 1 mL) were collected for serum from the brachial vein and then stored at −20°C for future analysis of SOD.

### 2.3. Contents of purine and its derivative analysis

The contents of purine (adenine, guanine, hypoxanthine, and xanthine) and uric acid in breast meat and liver were determined using high-performance liquid chromatography (HPLC) (Shimadzu modelLC20A, Tokyo, Japan). The breast meat and liver samples were minced separately, and approximately 500 mg of the samples were homogenized in 10 mL of deionized water containing 35% perchloric acid. The homogenate was incubated at 95°C, shaken at 180 rpm for 1 h in a water bath, and then immediately neutralized with 30% potassium hydroxide. The mixture was centrifuged at 3,500 × g for 15 min at 4°C. Finally, the supernatant was filtered through 0.45 μm filtration membranes and injected into an HPLC for analysis. The analytical column used in the experiment was an Asahipak GS-HQ 320HQ, 300 mm × 7.5 mm, 6 μm column (Showa Denko K.K., Tokyo, Japan) at a temperature of 35°C. HPLC was performed using a mobile phase of 150 mM sodium phosphate buffer (pH 2.5) at a flow rate of 0.6 mL min^−1^, and the running time was 35 min. All samples were measured twice, and the values were averaged. The total purine content was calculated from the combined amounts of each derivative (14, 46).

### 2.4. Superoxide dismutase analysis

The activity of the antioxidant enzyme SOD was determined following the instructions described by Ratchamak et al. (47). Briefly, 10 μL of plasma was mixed with 835 μL of a solution containing cytochrome C (1 mM) and xanthine (50 mM), and 155 μL of xanthine oxidase was diluted in sodium phosphate/EDTA buffer (50 and 100 mM, respectively, pH 7.8). Then, the absorbance was determined every 5 min in a spectrophotometer fitted with a temperature regulator maintained at 25°C. The concentration of xanthine oxidase was calculated to generate the optimum amount of O_2_^−^, with a consequent reduction of cytochrome C that was calculated as the rate of cytochrome C reduction of 0.025 units of absorbance/min (at a wavelength of 550 nm); the basis of this calculation is that 1 unit of total SOD activity corresponded to 50% of this value. Therefore, SOD activity in the sample decreased the rate of cytochrome reduction compared to the blank.

### 2.5. Statistical analyses

In order to check the normality of the data, the growth traits, purine content, and uric acid [calculated according to Kaneko et al. (14)] in breast meat and liver, and SOD in blood were subjected to the Shapiro–Wilk test. Levene’s test was used to assess the homogeneity of variance across treatments (chicken groups). Where a significant deviation from a normal distribution and/or homogeneity of variance was observed, the nonparametric Kruskal–Wallis ANOVA rank test was applied to determine the differences between the chicken groups (called treatments). Data (growth traits, purine content, uric acid, and SOD) were subjected to multifactor (sex, chicken hatch set, and breed group) analysis of variance (ANOVA) using a general linear model for unbalanced data (GLM procedure) of SAS package v.9.0. Where significant differences were detected, multiple pairwise comparisons were conducted using Scheffe’s test (*p* < 0.05). The treatment effects were significant at *p* < 0.05 using the Dwass–Steel–Critchlow–Fligner test. All data are expressed as the mean values ± standard deviations. For correlation analysis, the data of all three chicken groups were combined. Data (growth traits, purine contents, uric acid, and SOD) were analyzed by Pearson correlation coefficients using PROC CORR in the SAS program to characterize the relationship among variables, and the results were presented as a heatmap correlation separated by chicken group using the Microsoft Excel v.2021. After that, principal component analysis (PCA) for all data separated by breast meat and liver was used to analyze the intercorrelation. PCA was then used to extract the most important information from the data table, compress the size of the dataset by keeping only the important information, simplify the description of the dataset, and analyze the structure of the observations and the variables to express this information as a set of new orthogonal variables by displaying them as points in maps. The PCA dataset was further tested using the Kaiser Meyer-Olkin (KMO) measure of sampling adequacy. A KMO measure of 0.60 and above is considered adequate (48) using the factor program of the SPSS 22 statistical package.

## 3. Results

### 3.1. Comparison of growth traits

A comparison of body weight (BW), average daily gain (ADG), breast circumference (BrC), feed intake (FI), and feed conversion ratio (FCR) in purebred and crossbred Thai native chickens is presented in Table 1. Statistical differences between BW and ADG in all three chicken groups were found from 2 to 10 weeks of age (*p* < 0.05). The highest BW and ADG were found in 25%TN chickens (258.19 g and 36.83 g/day), followed by 50%TN chickens (163.50 g and 18.45 g/day) and 100%TN chickens (138.33 g and 15.60 g/day). For breast circumference traits, significant differences were found at 6, 8, and 10 weeks of age (*p* < 0.05). Crossbred chickens with 25%TN had the highest breast circumference (28.03, 31.12, and 36.62 cm), while purebred chickens had the lowest circumference (19.64, 21.56, and 23.14 cm). For FI and FCR, the results were consistent with body weight and average daily gain (*p* < 0.05).

**Table 1:** Characteristics of growth traits of purebred and crossbred Thai native chickens (mean ± SD).

Trait	Chicken groups		
	100%TN	50%TN	25%TN
Body weight (BW; g)			
BW0	33.61 ± 3.05	36.17 ± 3.91	36.63 ± 2.96
BW2	138.33 ± 14.70^c^	163.50 ± 13.41^b^	258.19 ± 12.19^a^
BW4	340.15 ± 34.62^c^	416.71 ± 36.52^b^	833.31 ± 31.37^a^
BW6	605.76 ± 47.76^c^	745.42 ± 59.42^b^	1502.33 ± 44.80^a^
BW8	862.30 ± 84.29^c^	1108.83 ± 115.70^b^	2191.94 ± 157.87^a^
BW10	1146.12 ± 130.29^c^	1476.00 ± 204.58^b^	2881.55 ± 261.15^a^
Average daily gain (ADG; g/day)			
ADG0–2	15.60 ± 2.14^c^	18.45 ± 2.31^b^	36.83 ± 3.46^a^
ADG2–4	13.62 ± 1.14^c^	16.89 ± 1.40^b^	34.90 ± 1.77^a^
ADG4–6	15.28 ± 1.69^c^	19.68 ± 1.93^b^	38.33 ± 2.83^a^
ADG6–8	18.18 ± 3.06^c^	23.12 ± 2.66^b^	42.61 ± 2.18^a^
ADG8–10	15.44 ± 3.77^c^	20.40 ± 2.17^b^	39.47 ± 2.49^a^
Breast circumference (BrC; cm)			
BrC6	19.64 ± 0.79^c^	21.35 ± 0.68^b^	28.03 ± 0.43^a^
BrC8	21.56 ± 0.72^c^	23.10 ± 0.73^b^	31.12 ± 0.70^a^
BrC10	23.14 ± 0.74^c^	26.86 ± 0.86^b^	36.62 ± 0.88^a^
Feed intake (FI; g/bird/day)			
FI0–2	16.44 ± 0.29^c^	18.35 ± 0.28^b^	20.03 ± 0.33^a^
FI2–4	31.20 ± 0.32^c^	35.10 ± 0.33^b^	40.12 ± 0.40^a^
FI4–6	42.77 ± 0.49^c^	48.35 ± 0.48^b^	55.03 ± 0.53^a^
FI6–8	50.56 ± 0.52^c^	55.10 ± 0.53^b^	63.12 ± 0.60^a^
FI8–10	58.30 ± 0.54^c^	70.86 ± 0.56^b^	80.62 ± 0.78^a^
Feed conversion ratio (FCR)			
FCR0–2	3.30 ± 0.06^c^	3.15 ± 0.05^b^	2.52 ± 0.03^a^
FCR2–4	2.66 ± 0.07^c^	2.60 ± 0.05^b^	2.22 ± 0.03^a^
FCR4–6	2.57 ± 0.05^c^	2.44 ± 0.04^b^	2.03 ± 0.02^a^
FCR6–8	2.23 ± 0.04^c^	2.10 ± 0.03^b^	1.88 ± 0.02^a^
FCR8–10	2.00 ± 0.04^c^	1.88 ± 0.03^b^	1.54 ± 0.02^a^

100%TN, purebred Thai native (TN) chickens; 50%TN, 50% Thai native with 50% broiler chickens; 25%TN, 25% Thai native with 75% broiler chickens; BW0, birth weight, BW2, BW4, BW6, BW8, and BW10, body weight at 2, 4, 6, 8, and 10 weeks of age; ADG0–2, ADG2–4, ADG4–6, ADG6–8, and ADG8–10, average daily gain during 0–2, 2–4, 4–6, 6–8, and 8–10 weeks of age; BrC6, BrC8, and BrC10, breast circumference at 6, 8, and 10 weeks of age; FI0–2, FI2–4, FI4–6, FI6–8, and FI8–10, feed intake during 0–2, 2–4, 4–6, 6–8, and 8–10 weeks of age; FCR0–2, FCR2–4, FCR4–6, FCR6–8, and FCR8–10, feed conversion ratio during 0–2, 2–4, 4–6, 6–8, and 8–10 weeks of age. ^a,b,c^Different superscript letters differed significantly between chicken groups (within each row) at *P* < 0.05.

### 3.2. Comparison of purine content, uric acid, and SOD

Purine content and uric acid in breast meat and liver and SOD in the blood of purebred and crossbred Thai native chickens are shown in Table 2. The quantity of purine content and uric acid in the liver were higher than in breast meat in all chicken groups, except hypoxanthine, which was more abundant in breast meat than in the liver. The lowest total purine, adenine, guanine, xanthine, hypoxanthine, and uric acid levels were found in 100%TN chickens, and the highest values were found in 25%TN chickens (*p* < 0.05). However, the purine content and uric acid in 100%TN and 50%TN chickens were not significantly different (*p* > 0.05). In addition, the highest values for each parameter and each chicken group were found at 6 weeks of age. Afterward, values for each parameter decreased with age. For SOD, the results were in the same direction as the results of purine content and uric acid; 25%TH had the highest SOD, while 100%TH had the lowest SOD.

**Table 2:** Characteristics of purine content and uric acid in breast meat and liver and SOD in the blood of purebred and crossbred Thai native chickens (mean ± SD).

Trait	Breast meat			Liver		
	100%TN	50%TN	25%TN	100%TN	50%TN	25%TN
Total purine (mg/100 g)						
6 week	150.41 ± 6.58^b^	161.67 ± 6.11^b^	178.37 ± 6.74^a^	315.58 ± 12.41^b^	347.59 ± 12.56^b^	383.15 ± 13.80^a^
8 week	146.75 ± 5.33^b^	158.97 ± 5.20^b^	172.93 ± 5.12^a^	301.11 ± 11.64^b^	322.36 ± 12.34^b^	356.16 ± 13.15^a^
10 week	142.13 ± 5.81^b^	151.49 ± 5.61^b^	161.68 ± 5.00^a^	279.84 ± 11.56^b^	289.63 ± 11.30^b^	327.40 ± 13.28^a^
Adenine (mg/100 g)						
6 week	30.30 ± 1.91^b^	35.47 ± 1.55^b^	39.77 ± 1.10^a^	108.88 ± 6.56^b^	125.93 ± 6.59^b^	137.51 ± 6.21^a^
8 week	29.55 ± 1.88^b^	33.63 ± 1.06^b^	38.33 ± 1.93^a^	101.54 ± 5.55^b^	114.75 ± 5.55^b^	131.96 ± 6.03^a^
10 week	27.23 ± 1.95^b^	30.23 ± 1.44^b^	34.63 ± 1.49^a^	94.25 ± 5.17^b^	101.73 ± 5.90^b^	121.38 ± 6.40^a^
Guanine (mg/100 g)						
6 week	33.03 ± 1.15^b^	36.27 ± 1.41^b^	42.55 ± 2.55^a^	135.44 ± 8.67^b^	143.30 ± 8.27^b^	159.85 ± 9.92^a^
8 week	32.25 ± 1.24^b^	36.90 ± 1.44^b^	40.70 ± 2.66^a^	131.42 ± 6.34^b^	135.83 ± 9.43^b^	145.60 ± 9.34^a^
10 week	33.17 ± 1.26^b^	34.13 ± 1.67^b^	38.95 ± 2.09^a^	129.13 ± 7.43^b^	127.40 ± 6.08^b^	138.84 ± 9.95^a^
Xanthine (mg/100 g)						
6 week	1.83 ± 0.58^b^	1.90 ± 0.25^b^	3.60 ± 0.75^a^	55.88 ± 3.34^b^	60.43 ± 3.95^ab^	66.54 ± 3.93^a^
8 week	1.70 ± 0.44^b^	1.87 ± 0.55^b^	3.20 ± 0.56^a^	50.72 ± 4.42^b^	56.20 ± 3.27^ab^	59.80 ± 3.92^a^
10 week	1.63 ± 0.31^b^	1.75 ± 0.47^b^	2.90 ± 0.43^a^	43.22 ± 3.43^b^	48.25 ± 3.08^ab^	51.38 ± 3.95^a^
Hypoxanthine (mg/100 g)						
6 week	85.25 ± 4.39^b^	88.03 ± 4.23^b^	92.45 ± 4.35^a^	12.38 ± 3.07^b^	17.93 ± 2.42^a^	19.25 ± 2.15^a^
8 week	83.35 ± 4.06^b^	86.57 ± 4.35^b^	91.70 ± 4.84^a^	10.43 ± 2.25^b^	15.58 ± 2.11^a^	18.80 ± 2.30^a^
10 week	80.40 ± 3.43^b^	81.78 ± 2.54^b^	86.20 ± 3.47^a^	9.24 ± 2.20^b^	12.25 ± 2.15^a^	15.80 ± 2.83^a^
Calculated as uric acid (mg/100 g)						
6 week	181.74 ± 4.47^b^	195.29 ± 4.26^b^	214.96 ± 5.74^a^	366.45 ± 5.82^c^	404.97 ± 5.75^b^	446.17 ± 6.82^a^
8 week	177.34 ± 3.28^b^	191.86 ± 4.82^b^	208.64 ± 5.61^a^	349.16 ± 5.07^c^	375.18 ± 5.09^b^	415.41 ± 6.07^a^
10 week	171.53 ± 3.47^b^	183.04 ± 4.73^b^	194.96 ± 4.98^a^	324.47 ± 5.17^c^	336.71 ± 5.94^b^	381.72 ± 5.17^a^
SOD (U/mL)						
	100%TN		50%TN		25%TN	
6 week	7.08 ± 0.58^b^		8.47 ± 0.44^b^		8.93 ± 0.45^a^	
8 week	6.49 ± 0.21^b^		6.77 ± 0.50^b^		7.71 ± 0.51^a^	
10 week	4.88 ± 0.30^b^		5.81 ± 0.40^b^		6.95 ± 0.38^a^	

Calculated as uric acid (mg/100 g) = $\frac{\mathrm{Total}\;\mathrm{purine}\;\left(\mathrm{\mu mol}/100g\right)\times 168.1}{1,000}$ when the molecular weights of adenine, guanine, xanthine, hypoxanthine, and uric acid = 135.1, 151.1, 136.1, 152.1, and 168.1, respectively. Converted purine content from mg to μmol = $\frac{\mathrm{amount}\;\mathrm{in}\;\mathrm{mg}/\mathrm{molecular}\;\mathrm{weight}}{1,000}$ . ^a,b,c^Different superscript letters differed significantly between chicken groups (within each row) separated by breast meat and liver at *P* < 0.05.

### 3.3. Correlations between purine content, uric acid, SOD, and growth traits

**Figure 1:**
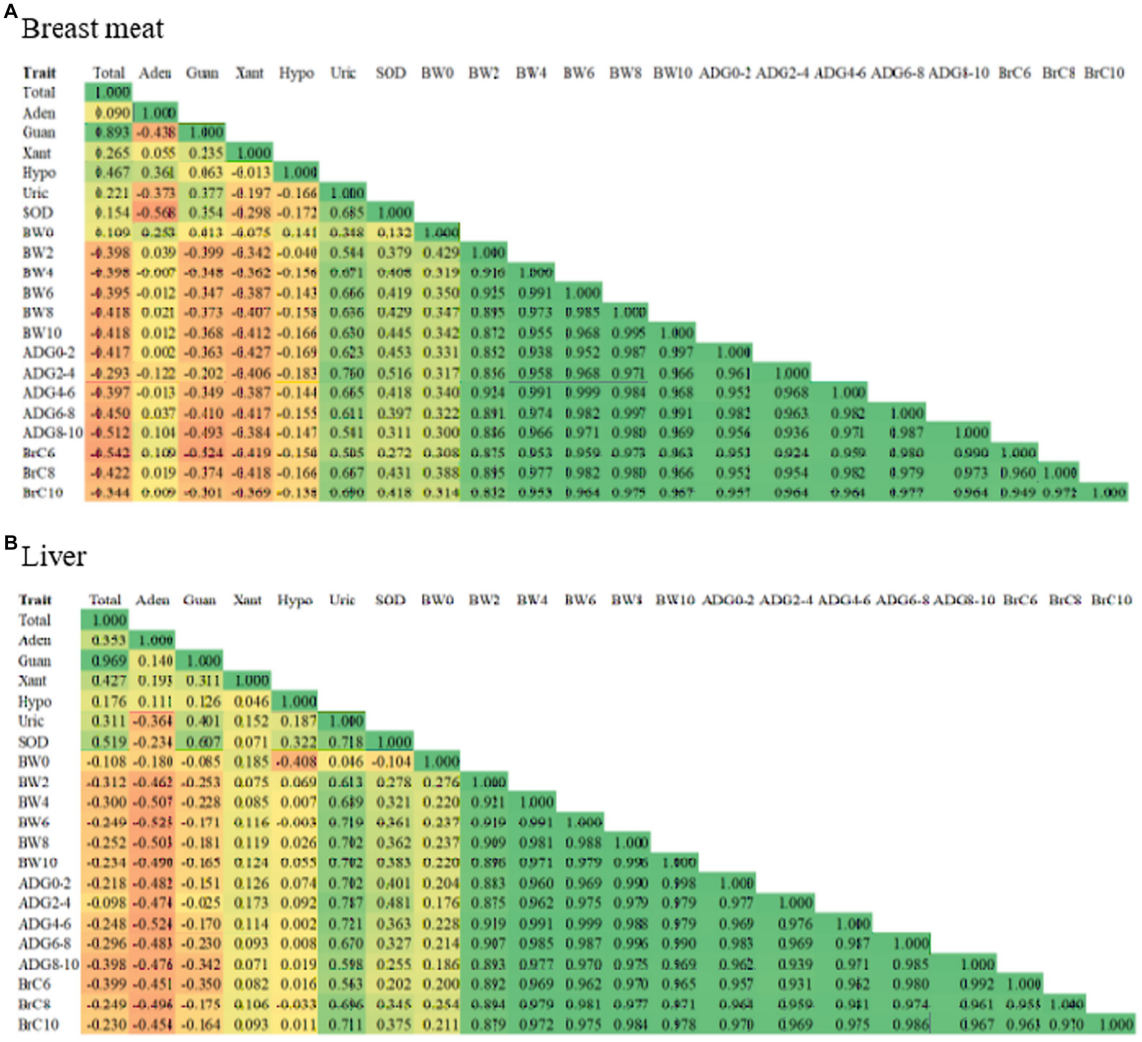
Heatmap illustrating correlation coefficient between purine content, uric acid, SOD, and growth traits in **(A)** breast meat, and **(B)** liver of purebred and crossbred Thai native chickens. Orange color means negative correlation, whereas green color represents positive correlation. Total, total purine; Aden, adenine; Guan, guanine; Xant, xanthine; Hypo, hypoxanthine; Uric, uric acid; SOD, superoxide dismutase; BW0, birth weight, BW2, BW4, BW6, BW8; BW, body weight at 2, 4, 6, 8, 10 weeks of age; ADG0–2, ADG2–4, ADG4–6, ADG6–8, ADG8–10, average daily gain during 0–2, 2–4, 4–6, 6–8, 8–10 weeks of age; BrC6, BrC8, BrC10, breast circumference at 6, 8, 10 weeks of age.

Correlation coefficients between purine content, uric acid, SOD, and growth traits are illustrated in Figure 1 using a heatmap. In the breast meat (Figure 1A), the correlations between total purine and uric acid and total purine and SOD were low positive correlations with values of 0.221 and 0.154, respectively, with significant differences (*p* < 0.05). However, when considering the contents of purine individually, low to moderate negative correlations were found between adenine, xanthine, and hypoxanthine and uric acid (−0.373, −0.197, and − 0.166) and SOD (−0.568, −0.298, and − 0.172) (*p* < 0.05), except correlations between guanine and uric acid (0.377) and guanine and SOD (0.354) were moderate positive correlations with significant differences (*p* < 0.05). The correlations between purine content (total purine, adenine, guanine, xanthine, and hypoxanthine) and growth traits (BW, ADG, and BrC) ranged from moderate negative to moderate positive (−0.542 to 0.253) with significant differences (*p* < 0.05). The correlations between uric acid and growth traits (0.348–0.760) and SOD and growth traits (0.132–0.516) were low to moderate positive with significant differences (*p* < 0.05). The correlation between uric acid and SOD was 0.685, with significant differences (*p* < 0.05). Finally, the correlations between and within BW, ADG, and BrC traits were highly positive and significant (*p* < 0.05). For the correlation in the liver (Figure 1B), it was found that most correlations between traits and within traits were in the same relationship as those found in the breast meat (*p* < 0.05), except for the correlations between growth traits and adenine, xanthine, and hypoxanthine, which were opposite in breast meat (negative correlations and significant, *p* < 0.05).

### 3.4. Principal component analysis results

**Figure 2:**
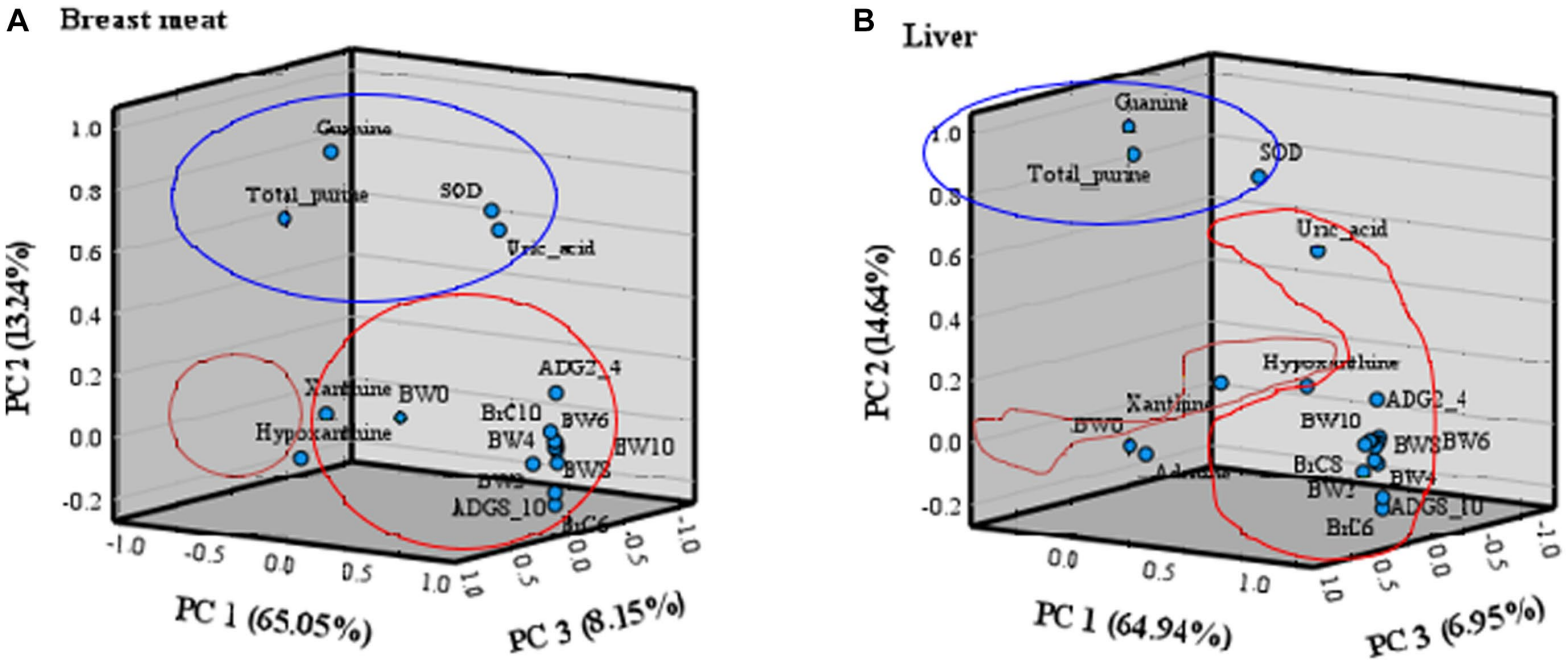
Three-dimensional component plot of purine content, uric acid, SOD, and growth traits in **(A)** breast meat and **(B)** liver by principal component analysis; PC 1 (red group), PC 2 (blue group), and PC 3 (orange group).

The principal component (PC) plot of purine content and uric acid in breast meat and liver, SOD in blood, and growth traits is presented in Figure 2. The statistical analysis highlighted three principal components (PC1, PC2, and PC3), explaining 86.44% in breast meat (Figure 2A) and 86.53% in liver (Figure 2B) of the total information. The PC plot showed clusters of samples based on their similarity, which provided a good separation between purine content, uric acid, SOD, and growth traits. In breast meat (Figure 2A), the first principal component (PC1) accounted for 65.05% (eigenvalue = 13.04) of the total variance and was related to the overall 14 growth traits (BW, ADG, and BrC). The second component (PC2) contributed to 13.24% (eigenvalue = 2.79) of the total variance and was related to total purine, guanine, uric acid, and SOD. The third component (PC3) accounted for 8.15% (eigenvalue = 1.86) of the total variance and was related to xanthine and hypoxanthine. In the liver (Figure 2A), PC1 accounted for 64.94% (eigenvalue = 13.64) of the total variance and was related to uric acid and all growth traits (BW, ADG, and BrC), except birth weight (BW0). PC2 contributed 14.64% (eigenvalue = 3.08) to the total variance and was related to total purine, guanine, and SOD. PC3 accounted for 6.95% (eigenvalue = 1.46) of the total variance and was related to birth weight and hypoxanthine. Adenine and xanthine were not categorized in PC1 to PC3.

## 4. Discussion

This study provides researchers with more genetic information about the relationship between bioactive compounds, antioxidant enzymes, and growth traits in Thai native chickens and its crossbred, which is the first to be reported. Purine-free diets do not exist; therefore, consuming high-purine chicken meat may have disadvantages and clinical risks, especially in individuals susceptible to gout, hyperuricemia, and kidney stones. Also, this findings of our study can be applied in chicken breeding programs (selected low purine content genetic line) for the development and genetic improvement of purebred and crossbred native chickens to have good growth characteristics and be suitable for developing a healthy food from chicken meat.

For growth traits, the 25%TN (crossbred) group had the highest growth traits compared to the 50%TN (crossbred) and 100%TN (purebred) groups, which were expected results. This is due to the genes of broiler chickens in large quantities resulting in 25%TN crossbred native chickens having average body weight, average daily gain, and breast circumference in each age range being the highest. Raising crossbred native chickens like those in the 25%TN group will increase the production cycles per year by two times the rearing time of purebred native chickens. However, even though the chickens in the 100%TN (Pure Thai native chicken) group had an average body weight, average daily gain, and breast circumference that were the lowest, their BW8 was better than other Thai native chicken breeds, such as Lueng Hang Kao Kabinburi (49) at 642.08 g at 8 weeks of age, and Pradu Hang dum (1) at 808.01 g at 8 weeks of age. Importantly, this study demonstrates that the genetic improvement of growth traits is more accurate and better than those studied in other Thai native chicken breeds. For purine content and uric acid, the results in this study were consistent with other studies; for example, Potue et al. (13) found that the total purine and purine contents (adenine, guanine, and hypoxanthine) of commercial broilers were 15 and 41% higher than those of purebred and crossbred Thai native chickens (Pradu Hang dum), respectively. Meanwhile, Kaneko et al. (14) and Hou et al. (50) showed that the purine content in chicken liver was more than twice as high as that in chicken breast meat. In addition, a study by Hou et al. (50) showed that the purine content in chicken liver was greater than that in other parts. In addition, Kubota et al. (46) showed that the purine content decreased with age in Korat chickens as found in this study.

A positive relationship between purine content and uric acid is biologically significant because elevated purine content is often converted to higher uric acid levels (51, 52). Also, higher purine content and uric acid in the chicken liver compared with chicken breast meat can be attributed to several factors. For instance, the liver is responsible for various metabolic and recycling processes, including the breakdown of purine into uric acid. As a result, the liver contains higher concentrations of purines and their metabolic byproduct, uric acid (53). The diet of the chicken can also influence the purine content in its organs. Chickens that consume foods rich in purines, such as insects or plant materials high in purine content, may have higher purine levels in their liver than chickens with a different diet (51, 52). Hou et al. (50) showed that one-third of uric acid is produced exogenously and two-thirds endogenously. Different tissues of an animal can have varying compositions. The chicken liver is a highly metabolic organ with a unique cellular structure compared to chicken breast meat (54, 55). The liver contains higher concentrations of nucleic acids, which are purine-rich compounds, contributing to the overall purine content. Moreover, purebred Thai native chickens are considered to be slow-growing chickens (100%TN) with lower purine and uric acid levels than crossbred Thai native chickens, which are classified as fast-growing chickens (25%TN and 50%TN). This is due to the following factors. Firstly, fast-growing chickens are bred to have rapid muscle growth and high feed efficiency (56, 57). Their metabolism is geared toward quick energy production and protein synthesis, which can result in increased purine metabolism (7, 19, 20, 58). This elevated metabolic rate can lead to higher purine turnover and, subsequently, higher uric acid levels in the body. Secondly, slow-growing chicken breeds are generally selected for different traits compared to fast-growing breeds. The genetic makeup of slow-growing chickens may result in lower purine production or less purine metabolism, leading to reduced purine and uric acid levels (59). It is important to note that while slow-growing chickens may have lower purine and uric acid levels, the total purine in chicken meat is not exceptionally high (<200 mg/100 g) (13, 50).

As chickens age, their metabolic rate slow down, and their organs, including the liver and kidneys, become less efficient. To compensate for this, older chickens naturally produce lower levels of purines, resulting in lower uric acid levels in their bodies. This is thought to occur because the enzymes involved in purine synthesis and metabolism are downregulated or inhibited in older chickens, thereby reducing the overall production of purines and uric acid. Additionally, older chickens may also have changes in their gut microbiome, which can affect the metabolism of purines and other compounds. For example, changes in the gut microbiome can lead to a reduction in the availability of certain nutrients, which can, in turn, affect the synthesis of purines and other compounds. Overall, the metabolic mechanisms that result in lower levels of purines and uric acid in older chickens are complex and multifactorial and involve changes in enzyme activity, gut microbiome composition, and overall metabolic rate.

In the study of relationships, understanding correlation heatmaps can help us identify patterns and relationships between multiple variables. The positive relationship between total purine and uric acid content in chicken meat can be attributed to the fact that uric acid is a purine metabolite. When purines are broken down in the body, one of the end products is uric acid. Therefore, a higher concentration of purines in chicken meat would naturally result in a higher concentration of uric acid (13, 21, 22). Chickens, like humans and many other animals, metabolize purines into uric acid. The metabolic process involves the breakdown of dietary purines and endogenous purines (nucleic acids) within the chicken’s body. The uric acid formed during this process is excreted via the kidneys. An imbalance in purine metabolism or excretion can lead to elevated uric acid levels in meat. When the purine and uric acid levels in chicken meat increase significantly, several problems can occur within the chicken’s body, such as metabolic stress on the chicken’s body as it tries to process and eliminate excess purines, imbalance in the chicken’s immune system, more susceptible to infections and diseases causing inflammation, discomfort, and impaired mobility, finally reduced growth rates and decreased overall productivity. In addition, the synthesis of fatty acids, especially triglycerides, in the liver is associated with the *de novo* synthesis of purine, accelerating uric acid production (19, 20, 60).

The relationship between total purine and superoxide dismutase (SOD) activity in chicken meat may exhibit a positive correlation due to the antioxidant properties of purines and the role of SOD in the antioxidant defense system. Purines, including adenine and guanine, possess antioxidant properties (61). They can act as scavengers of free radicals and reactive oxygen species (ROS) that are generated during cellular metabolism. ROS can cause oxidative damage to cells and tissues (62). The presence of purine content in chicken meat with higher total purine content could indicate a higher antioxidant capacity, potentially helping to counteract oxidative stress (63). The positive correlation between total purine content and SOD activity in chicken meat could indicate that higher purine levels contribute to an enhanced antioxidant defense system. This suggests that chicken meat with higher total purine may exhibit increased levels of SOD activity, helping to neutralize ROS and reduce oxidative stress. It is important to note that various factors, including genetics, diet, and environmental conditions, can influence both purine content and SOD activity in chicken meat. Additionally, other antioxidant enzymes and compounds may also play a role in the overall antioxidant capacity of the meat. Therefore, the relationship between total purine and SOD activity should be considered within the broader context of antioxidant defenses in chicken meat.

The relationship between uric acid and SOD can be both positive and negative relationship, as the analysis suggests, because the two variables are very different in their mechanisms of action. Uric acid is a natural antioxidant present in the blood and tissues of humans and certain animals, including chickens. It acts as a scavenger of free radicals and reactive oxygen species (ROS), protecting cells from oxidative damage (64). Uric acid can directly neutralize certain ROS and indirectly contribute to the antioxidant defense system. At the same time, SOD is an enzyme that plays a vital role in the antioxidant defense system by catalyzing the conversion of superoxide radicals into less harmful molecules (65). SOD helps neutralize superoxide radicals, reducing oxidative stress and potential damage to cells. While both uric acid and SOD are involved in antioxidant processes, their relationship may not necessarily exhibit a consistent positive correlation in chicken meat. Several factors can influence the relationship between uric acid and SOD activity, including gene expression, enzymatic regulation, and cellular signaling pathways. The levels of SOD activity can be influenced by various factors, such as diet, genetic factors, and environmental conditions. These factors may not necessarily align with the levels of uric acid present in chicken meat. Although uric acid and SOD represent different components of this system, while they both contribute to the overall antioxidant capacity, their relationship can be influenced by other antioxidant enzymes and molecules present in chicken meat.

The relationship between purine content and growth characteristics is consistent due to the fundamental role that purine content plays in various biological processes. Conservation of basic cellular processes: purine content is involved in fundamental cellular processes such as DNA replication, RNA synthesis, and protein synthesis (16, 17). These processes are highly conserved across living organisms, from bacteria to plants and animals (66). The need for purine content in these processes ensures that they remain crucial for growth and development throughout the evolutionary spectrum (67). Biochemical pathways: purine content is synthesized through a series of enzymatic reactions in organisms. These pathways are conserved across species, ensuring the production of purine content for essential cellular functions (68). The consistency in purine biosynthesis pathways contributes to the consistent relationship between purine content and growth traits. Regulatory mechanisms: purine metabolism and utilization are tightly regulated in cells. Feedback mechanisms and regulatory enzymes control the levels of purine content to maintain the balance between synthesis and degradation (15). This regulation ensures that adequate purine levels are available for growth-related processes while preventing excessive accumulation or depletion (69). The consistency in these regulatory mechanisms contributes to the consistent relationship between purines and growth traits. Evolutionary significance: efficient utilization of resources is critical for the survival and reproduction of organisms (70). Purine content, which is vital for growth and development, has likely been under strong evolutionary pressure to maintain a consistent relationship with growth characteristics (71). Organisms that can effectively synthesize, regulate, and utilize purine content are more likely to exhibit consistent growth traits, providing them with a selective advantage. While the relationship between purine content and growth characteristics is generally consistent, it is important to recognize that there can still be variations due to genetic factors, environmental influences, and specific physiological contexts. Nonetheless, the central role of purines in critical cellular processes ensures a consistent relationship with growth traits across different organisms.

The principal component (PC) analysis technique can help to elucidate the relationship of various traits in growth characteristics (BW, ADG, and BrC), purine content, uric acid, and SOD in purebred and crossbred Thai native chickens. The PC plot expressed how the same PC explained correlated variables, and less correlated variables were explained by different PCs (72, 73). The results found this time, the PC1 was primarily associated with growth traits, with a positive correlation between traits evident from body weight at 2 weeks of age onwards. Thus, when choosing growth traits to analyze the selection indexes, one trait of body weight at 2 weeks (BW2) may be sufficient for evaluating growth genetics. It also enables faster animal selection without waiting for growth data to be recorded until maturity. The PC2 is related to total purine, guanine, uric acid, and SOD, with a mutually positive correlation in each trait. It is worth noting that guanine data collection can very well describe changes in total purine, uric acid, and SOD and can save time and budget for other data collection. For PC3, the relation to xanthine and hypoxanthine in both chicken breast meat and liver is unclear due to its low relationship; therefore, in the authors’ opinion, it is suggested that selective use of PC1 and PC2 is sufficient to explain the relationship between growth characteristics, bioactive compounds, and antioxidants to stress. The results led to an objective simultaneous analysis of these growth traits and bioactive compounds rather than an individual analysis of each. Additionally, the use of three-dimensional components (PC1, PC2, and PC3) was more appropriate than the use of the original interrelated parameters for predicting the growth characteristics and bioactive compounds of native chickens. In addition, three principal components in breast meat and liver could aid in selection and breeding programs in terms of the selection index and genetic evaluation (74) of native chickens. The present findings are consistent with those reported by Pinto et al. (75) in broilers, Yakubu et al. (76) in Nigerian indigenous chickens, and Mussa et al. (77) in Tanzanian indigenous chickens, where PC1 was termed overall body size, general weight, and meat quality. However, despite the potential benefit given by principal components for multitrait evaluation, its use in animal breeding programs has been relatively scarce, perhaps partly because it is not always possible to explain high variance percentages with few principal components, as occurred in this study. However, in circumstances when a good adjustment occurs, its practical use can bring great benefits to animal breeding programs because it can greatly facilitate evaluations. Principal component analysis is an interesting tool for evaluating and understanding the total variance originating in a group of correlated traits, allowing for a drastic reduction in the number of traits to be considered in the selection index of poultry breeding programs. The selection of animals for any principal component does not cause correlated responses in terms of other principal components (75). This is buttressed by the report of Yamaki et al. (78), who used principal component analysis to identify independent and informative variables, thereby eliminating redundant information for the purpose of reducing the costs of quail genetic programs.

In conclusion, purine content, uric acid, and SOD of Thai native chickens differed depending on the genetic make-up of Thai chickens’ breed groups ranging from purebred to their crossbreds. We found from our study that increased levels of purine and uric acid are associated with fast and improved growth performance in Thai chicken Crossbreds than in pure Thai chickens. However, exaggerated levels of purines and uric acid in chicken may permit negative biological consequences, such as metabolic stress, which may lead to the accumulation of more free radicals; this may cause an imbalance in the immune system, susceptibility to infections and disease conditions, reduced growth rates, and decreased overall productivity. The development of improved growth characteristics in chicken leads to an increase in uric acid which subsequently increases SOD values in terms of antioxidant potentials (scavenging of free radicals). Earlier studies concluded that consuming high-purine chicken meat may pose public health challenges and clinical risks, especially in individuals susceptible to gout, hyperuricemia, and kidney stones. Finally, this study provides biological information to livestock farmers, particularly for poultry farmers, particularly Thai farmers to produce crossbred native chickens with faster production cycles.

## Data availability statement

The original contributions presented in the study are included in the article/supplementary material, further inquiries can be directed to the corresponding author.

## Ethics statement

The animal studies were approved by the Institutional Animal Care and Use Committee, based on the Ethics of Animal Experimentation of the National Research Council of Thailand (record no. IACUC-KKU-14/65). The studies were conducted in accordance with the local legislation and institutional requirements. Written informed consent was obtained from the owners for the participation of their animals in this study.

## Author contributions

WB: Funding acquisition, Methodology, Project administration, Writing – original draft, Writing – review & editing, Conceptualization, Data curation, Formal analysis. VT: Conceptualization, Data curation, Formal analysis, Methodology, Writing – original draft. KC: Conceptualization, Data curation, Writing – original draft. WL: Writing – original draft. VC: Formal analysis, Funding acquisition, Project administration, Writing – original draft.
